# *Kerteszia *subgenus of *Anopheles *associated with the Brazilian Atlantic rainforest:current knowledge and future challenges

**DOI:** 10.1186/1475-2875-6-127

**Published:** 2007-09-19

**Authors:** Mauro Toledo Marrelli, Rosely S Malafronte, Maria AM Sallum, Delsio Natal

**Affiliations:** 1 Departamento de Epidemiologia, Faculdade de Saúde Pública, Universidade de São Paulo, Avenida Dr. Arnaldo 715, São Paulo-SP, 01246-904, Brazil; 2 Laboratório de Protozoologia, Instituto de Medicina Tropical de São Paulo, Universidade de São Paulo, Avenida Dr. Enéas de Carvalho Aguiar, 470, São Paulo-SP, 05403-000, Brazil; 3 Departamento de Doenças Infecciosas e Parasitárias, Faculdade de Medicina, Universidade de São Paulo, Avenida Dr. Arnaldo, 455, São Paulo-SP, 05403-000, Brazil

## Abstract

**Background:**

The Atlantic rainforest ecosystem, where bromeliads are abundant, provides an excellent environment for *Kerteszia *species, because these anophelines use the axils of those plants as larval habitat. *Anopheles (K.) cruzii *and *Anopheles (K.) bellator *are considered the primary vectors of malaria in the Atlantic forest. Although the incidence of malaria has declined in some areas of the Atlantic forest, autochthonous cases are still registered every year, with *Anopheles cruzii *being considered to be a primary vector of both human and simian *Plasmodium*.

**Methods:**

Recent publications that addressed ecological aspects that are important for understanding the involvement of *Kerteszia *species in the epidemiology of malaria in the Atlantic rainforest in the Neotropical Region were analysed.

**Conclusion:**

The current state of knowledge about *Kerteszia *species in relation to the Atlantic rainforest ecosystem was discussed. Emphasis was placed on ecological characteristics related to epidemiological aspects of this group of mosquitoes. The main objective was to investigate biological aspects of the species that should be given priority in future studies.

## Background

The Atlantic rainforest originally stretched as a continuous domain from northeastern to southern Brazil, northern Argentina and southeastern Paraguay. The forest occupied a narrow coastal area in northeastern Brazil, but in some parts of the state of São Paulo, it extended from the coast to as far as 200 miles inland [1].

Deforestation started during the colonization of Brazil in the XVI century. As a result, most of the forest has been cleared, and currently less than 7.6% of its original 1,306,000 km2 cover remains, but is highly fragmented. The forest domain persists in a relatively continuous area in mountainous regions and as small fragments in regions with a smooth topography, which are generally used for agriculture [2].

Apart from cases of *Anopheles neivai *reported in the Amazon region and Iguassu Park, where *Anopheles cruzii *can also be found [3], the geographic domain of *Kerteszia *species is restricted to those areas of the Serra do Mar mountains that remain covered with the exuberant Atlantic forest. The humid and rainy climate, together with the rocks and soil, support several species of bromeliads.

Since the subgenus was described, the association between *Kerteszia *immature stages and bromeliads has been observed by various authors. *Anopheles bambusicolus *is the only species that uses bamboo as larval habitat. The massive presence of bromeliads in both Serra do Mar mountains and the coastal plain near this mountain range is indicative of the occurrence of *Kerteszia *species. Areas where *Kerteszia *species occur are considered risk areas for *Plasmodium *transmission.

It is noteworthy that malaria epidemics constituted a challenge to public health authorities in the past [4], mainly in areas along the Serra do Mar mountain range, from the south of the state of São Paulo to the state of Santa Catarina. Following the decline in malaria transmission in these states, the disease now persists at low endemic level and the transmission area has extended to the northeast Brazil. Currently, few malaria cases have been notified. In the state of Espírito Santo, malaria continues to be a concern to epidemiologists and the public health surveillance service [5].

Previous reviews of the literature on *Kerteszia *have focused on systematics and taxonomy [6], simian malaria [7] and historical aspects [8]. This review was undertaken in view of the situation described above and the relevance of the *Kerteszia *subgenus. It focuses on both the current state of knowledge and ecological aspects of this taxon. It is hoped that a critical analysis of the literature and the experience of those working in this field will help identify which studies should be given priority in the coming years. The review covers the following subjects: geographic distribution, taxonomy, habitat, behaviour and the relationship of *Kerteszia *species with the transmission and genetic makeup of malaria parasites. Given that *An. cruzii *and *Anopheles bellator *are involved in the dynamics of *Plasmodium *transmission in the Atlantic forest domains, these species are the major focus of this review.

### Geographic distribution and habitats

Maps of the geographic distribution of the *Kerteszia *species generated by Zavortink [6] show that this taxon is widely distributed throughout the Americas, from Mexico to southern Brazil. The majority of *Kerteszia *species occurs in coastal areas of both the Pacific and Atlantic oceans, where bromeliads are abundant These belong to the monocotyledons Bromeliaceae family, that accumulate water in leaf axils [9]. Hundreds of species of bromeliad have this characteristic and provide a suitable habitat for immature of mosquitoes, including those of the subgenus *Kerteszia*.

**Figure 1:**
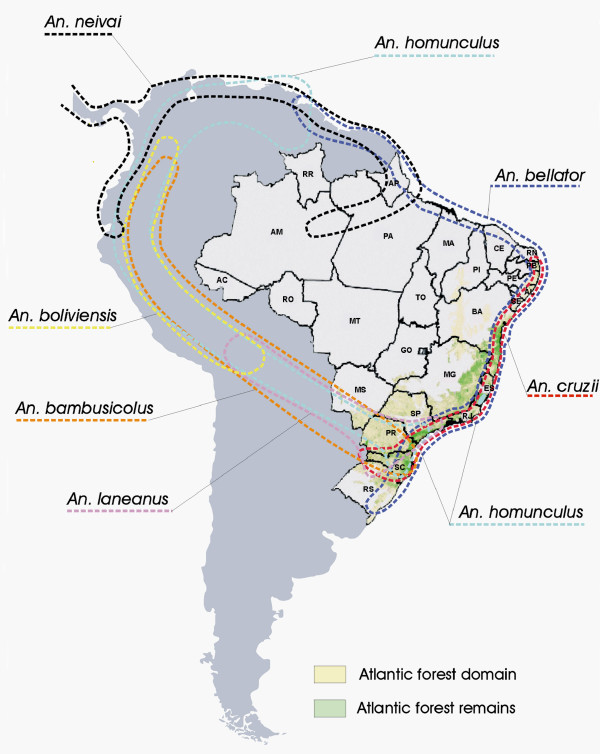
Geographic distribution of the *Kertezsia *species in South America. The Atlantic forest domain and the remains in Brazil are indicated. States citated in the text: AP – Amapá, BA – Bahia, ES – Espírito Santo, PB – Paraíba, RJ – Rio de Janeiro, SC – Santa Catarina, SP – São Paulo, SE: Sergipe)

Zavortink [6] recorded the presence of *An. cruzii*, *An. bellator*, *Anopheles homunculus*, *An. bambusicolus *and *Anopheles laneanus *in the Brazilian Atlantic rainforest. Of these species, only *An. cruzii *and *An. bellator *are of epidemiological importance. In agreement, they are the most widespread species, occurring from southern Brazil to the northeastern limit of the Atlantic rainforest (Figure 1).

According to Forattini [10], the distribution range of *An. cruzii *extends from the state of Sergipe to areas of southern Brazil. *Anopheles bellator *extends along the northeastern coast, reaching the state of Amapá, northern South America and Trinidad and Tobago. However, its distribution is discontinuous (Figure 1). Consoli and Lourenço-de-Oliveira [11] recorded this species in an area extending from southern Brazil to the state of Paraíba and in Guyana. The species is currently believed to be distributed in two areas, one in northern South America and the other in Brazil, where it is associated with the Atlantic forest domain.

The other species known to occur in the Atlantic forest in Brazil, are *An. homunculus*, found in the states of Santa Catarina, Paraná and São Paulo, and recently registered in mountain areas of Espírito Santo state (Malafronte *et al*, unpublished data) (Figure 1); *An. bambusicolus*, registered in the states of Santa Catarina and Paraná; and *An. laneanus*, which occurs in the states of São Paulo and Rio de Janeiro, in high altitudes [6,10,11] (Figure 1).

Two other *Kerteszia *species are known from Brazil, *Anopheles neivai *recorded from the Amazon region [12], and *Anopheles bolivienses *found in Brazil (unspecified localities) [13,14]. Although *An. boliviensis *had been recorded from Brazil, the presence of this species has not been registered recently. Further field collections will be necessary to ascertain the occurrence of *An. boliviensis *in Brazil.

Habitat studies carried out in mountainous areas close to the coastal plain in the municipality of Cananéia (state of São Paulo) reported a higher frequency of *An. cruzii *than in lowland areas in the same region [15]. *Anopheles bellator *was found on the coastal plain in a sunny "restinga" habitat, while *An. cruzii *was the most frequent species on the mountain slopes. In the Serra da Bocaina mountains, in municipalities located on the border between the states of Rio de Janeiro and São Paulo, *An. cruzii *was the most abundant mosquito at high altitudes [16].

Forest with bromeliad cover is a favourable habitat for *An. cruzii*. Guimarães *et al *[3] recorded an isolated population of *An. cruzii *in Iguassu National Park, where this species was found exclusively in preserved areas covered by dense forest.

### Environmental effect

Due to the fact that mosquitoes of the *Kerteszia *subgenus are concentrated in exuberant humid forests, where there is a high density of bromeliads [17], populations of these species can have an impact effect according to the degradation of the environmental.

Dorvillé [18] analysed the data of twenty Culicidae surveys by employing multivariate statistical procedures to look for an association between taxon and the extent of the degradation in the areas where the collections were made. This author concluded that mosquitoes of the *Kerteszia *subgenus can be used as bio indicators and that their presence reflects the high degree of environmental preservation.

Because of the association between *Kerteszia *and bromeliads, Downs and Pittendrigh [19] coined the term bromeliad-malaria to describe malaria transmission vectored by these mosquitoes and to differentiate it from malaria transmitted by anophelines of the subgenera *Nyssorhynchus *and *Anopheles*, which breed in different water collections. *Anopheles cruzii*, *An. bellator *and *An. homunculus *are thus known bromeliad-malaria vectors in southern and southeastern Brazil [14,19-21] (more details, see *Plasmodium *transmission section).

Bromeliad-malaria was a serious public health problem in Brazil during the nineteenth century and the first half of the twentieth century [8]. The close contact between humans and the Atlantic rainforest led to extensive exposure of humans to mosquito bites and bromeliad-malaria transmission during those periods. Because bromeliads require a forest habitat, the deforestation caused by man has decimated these plants and thus drastically reduced the number of mosquitoes of this subgenus. This was observed by Forattini *et al *[22] in the Ribeira Valley, SP, when they compared the faunas of primitive forest in the quaternary plain and transition area with those in an artificially modified environment in the same region. The control measures implemented in the 1940s, which included deforestation and elimination of bromeliads, together with the use of chemical insecticides and anti-malarial drugs, resulted in a significant decrease in the number of malaria cases in the Atlantic forest region (from 40,000 in 1940 to only 71 in 1982) [23].

However, the Atlantic forest region has became preserved by environmental legislation and deforestation and elimination of bromeliads have been forbidden. Thus, despite the control measures that were implemented in the past, malaria has not been eradicated in southern Brazil. In the state of São Paulo, autochthonous cases of malaria are reported every year in the Atlantic Coast region and Ribeira Valley. In the State of Santa Catarina, *Plasmodium *transmission still occurs in some municipalities [24]. The present situation, in which the malaria parasite and vectors coexist, demands constant surveillance from public health authorities to prevent epidemic episodes.

*Anopheles cruzii *was the most frequent anopheline in the Serra da Cantareira mountains around the city of São Paulo in the 1960s, where it was demonstrated to be a vector of *P. simium*. Interestingly, a case of human malaria that occurred among the participants of the field collections was attributed to the same vector [7,25]. At that time these authors observed a high prevalence of this species in the canopy level of the forest. It is worth noting that Montes [26] studied the same forest, using CDC traps and dry ice in the canopy twelve meters from the ground at two-week intervals for one year, and failed to capture a single sample of this species. This suggests that the species may have been eliminated from the area. The authors questioned whether this was indeed the case and, if it was, what the reason for the disappearance of the species might have been. They hypothesised that the species might have disappeared because forest environmental in the Serra da Cantareira had suffered an intensive and constant human interference. Another possibility is that the elimination of the species was caused by the atmospheric pollution, which produces acid rain in the greater São Paulo. This phenomenon could have contaminated the bromeliads, making them unsuitable as habitat for the immatures of *Kerteszia*.

### Acrodendrophily

*An. cruzii *is significantly more active in the uppermost branchy layer of the forest than at ground level. In a review of simian malaria, Deane [7] reported that populations of *An. cruzii *from different locations were noticeably active in the canopy. According to Deane, in the Serra da Cantareira mountains on the outskirts of the city of São Paulo, where *Plasmodium simium *transmission to monkeys and humans were registered, *An. cruzii *constituted 94% of the total number of anophelines, and 99% were collected in the forest canopy.

Acrodendrophily of *An. cruzii *was confirmed in a study focusing on the vertical distribution of this species in the forest of the Serra dos Órgãos National Park, in the state of Rio de Janeiro [27]. Eighty-five percent of *An. cruzii *were captured on a 10-meter-high platform, and the remaining 15% collected at ground level.

Reproductive isolation between the canopy and ground-level populations was suspected by Deane *et al *[28]. To test this hypothesis, the authors performed a mark-release-recapture experiment to determine whether the mosquitoes shared the different levels of the forest. The experiment showed that there was only one population and that the mosquitoes flew between both levels.

### Biting activities

The females of the *Kerteszia *subgenus blood feed continuously during diurnal hours, mainly inside humid forests. According to Forattini *et al *[22], two peaks of activity can be identified: a high peak associated with the sunset and a secondary peak associated with sunrise, while Tubaki *et al *[29] described a night-time peak of activity for *An. cruzii *in Peruíbe, state of São Paulo.

*Kerteszia *populations in southern and southeastern Brazil have seasonal activity patterns defined by the climate, in particular the rainfall and temperature [29,30]. Thus, in hot and rainy seasons, which usually occur in the summer, the mosquito population density increases, while it decreases during the dry season and at lower temperatures [17]. However, there is no interruption in mosquito activity during the year. Forattini *et al *[15] observed in a primary forest in the Ribeira Valley that *An. cruzii *was active throughout the year, even in periods when species density was low.

Studies performed in well-preserved forest areas located near human modified areas showed that there is high affinity between *Kerteszia *mosquitoes and human beings. In a study carried out on the São Paulo State coast, Forattini *et al *[31] reported a high frequency of *An. cruzii *in the forest, while *An. bellator *was more abundant in the domestic environment. In the same study, *An. bellator *was found to have more endophagic and endophilic behaviour than *An. cruzii*.

The synanthropy of *Kerteszia *species was investigated in an island in the south of the state of São Paulo, where the topography is similar to that of the coastal plains and there is a low forest cover [32]. Using human-bait technique in the region extending from the forest to the area occupied by humans, the authors observed synanthropy of *An. cruzii *and *An. bellator*. This observation led to the interpretation that the females can fly to the anthropic environment to feed on blood. In the same study, negative synanthropy was detected when manual aspirators were used to capture mosquitoes in different resting places. The exophagic behaviour suggests that the females return to the natural environment after a blood meal. This behaviour was confirmed in a study carried out in Serra do Mar State Park by Guimarães *et al *[33]. Three different environments were explored, forest, agriculture and domestic. Consequently, *An. cruzii *was observed to blood feed in the domestic and peridomestic areas.

The physiological age (Parity) of the mosquitoes is also related to their biting activities. Studies of natural populations of *Kerteszia*, based in the Polovodova method [34] to determine the physiological age, indicated that *An. cruzii *and *An. bellator *are predominantly nulliparous, with low percentages of uniparous and even lower of biparous [30,35,36]. This low longevity would not explain the vector role of these species, mainly for the rarity of biparous females (0.1% or 0.0%), as reported in these publications. However, the same authors reported the presence of nulliparous females, captured using human baits, which ovaries were developed in stages of III the V of Christophers and Mer. These researchers interpreted this fact as an evidence of gonothrophic discordance in this taxon and that, therefore, the females can feed in more than one host to complete their gonothrophic cycle, increasing their vector potential. Regarding to this condition, it is admitted that, even so low longevity, the raised density of these mosquitoes constitutes preponderant factor for their vectorial performance.

### *Plasmodium *transmission

As previously mentioned, *An. cruzii *and *An. bellator *have long been known as important vectors of human and simian malaria parasites [14,25] in southeastern Brazil, mainly due to their biting behaviour discussed before. However, most cases occurred in patients coming from the Amazon region. Only a small number of autochthonous cases are notified every year in the Atlantic forest, and they are usually oligosymptomatic or even asymptomatic, with a very low parasitaemia [37].

Between 1990 and 2000, about 137 cases of autochthonous *Plasmodium vivax *malaria occurred in this region. These cases represented 90% of all the malaria cases in the state of São Paulo and are closely related to human activities. Morphologically, the parasites associated with autochthonous cases in the Atlantic forest have been diagnosed as *Plasmodium vivax*, but Deane [7] suggested that another species, such as simian *Plasmodium*, could be associated with "bromeliad-malaria".

Nowadays, *An. cruzii *mosquitoes are still involved in the transmission of human and simian *Plasmodium *in the valleys of the Atlantic rainforest in the states of Rio de Janeiro, Espírito Santo, São Paulo, Paraná and Santa Catarina [37-41] and were found, using the ELISA technique, to be infected with *P. vivax *and one of its variants, *P. vivax *VK247 [40].

The suspected relationship between human and non-human primate malaria is clearly evident in these regions. Serological studies revealed a high frequency of antibodies against peptides of circumsporozoite protein corresponding to *Plasmodium vivax *variants (VK210 and VK247), *P. malariae/P. brasilianum *and human *P. vivax*-like/*P. simiovale *in local human populations and in different wild monkey species. In addition, using the PCR technique, one inhabitant was found to be infected with *P. malariae*, suggesting that malaria could be considered a zoonosis among these inhabitants and that monkeys have been acting as malaria reservoirs in these non-endemic areas [37,41-43].

### Morphological characteristics

The subgenus *Kerteszia *comprises twelve species, most of which recorded from Venezuela. Morphological characters have been used to separate *Kerteszia *species, including those found in the Atlantic rainforest. Species identification, however, can be problematic. For example, to distinguish between *An. cruzii *and *An. homunculus *using only adult female characteristics, one needs well preserved specimens, because the identification is based on the colour of the pigments of the integument and presence, colour, size and position of the spots of pale scales on the maxillary palpomeres.

In considering *An. homunculus*, *An*. *laneanus*, *An. cruzii *and *An. bellator*, for an accurate species separation one needs male and female adults associated with fourth instar larva and pupa. Also, the specimens need to be well preserved and the male genitalia carefully dissected and mounted on microscope slide.

Morphological differences in the male genitalia have been widely used to distinguish species of *Anopheles *[6,44,45]. *Anopheles cruzii, An. bellator*, *An. homunculus *and *An. laneanus *can be separated by characteristics of the ventral and dorsal claspettes and the aedeagus.

Morphological comparisons of male genitalia of specimens identified as *An. homunculus *collected in areas of the Atlantic forest show that samples from Brazil can be distinguished from those illustrated by Komp [46] and Zavortink [6] by the shape of the setae on the dorsal claspette. Consequently, detailed studies using both morphology and molecular markers will be needed to ascertain the species status of *An. homunculus *found in the Atlantic Forest. We believe that the population that occur in the east side of Atlantic Forest may correspond to a not described species that has been largely misidentified as either *An. cruzii *or *An. homunculus*. Furthermore, if this is correct, the vector status of *An. cruzii *may be overestimated because *An. homunculus *may also be involved in malaria transmission in Atlantic Forest. These species are sympatric and have been collected in the same habitat (Sallum *et al*. unpublished data). Certainly, these hypotheses need to be tested with further studies. Additionally, characters of both fourth-instar larvae and pupae can distinguish *An. cruzii*, *An. laneanus*, *An. homunculus *and *An. bellator *(see [6,10,47] for details), however, details of the colour pattern of the whole fourth instar larva should be used to species separation because they clearly distinguish the species from Atlantic Forest (Sallum *et al*. unpublished data).

### Genetic studies

Because of the epidemiological importance of *An. cruzii *in *Plasmodium *transmission, studies of the morphological characteristics, genetic population and molecular polymorphism of *Plasmodium *vectors have focused on this species. These studies suggest that *An. cruzii *may constitute a species complex [6,48-51]. However, despite their epidemiological importance, little is known about the genetics of these anophelines.

Morphological differences were observed among populations from the states of Santa Catarina and Rio de Janeiro [6], suggesting that *An. cruzii *could represent more than one differentiated population. Ramirez and Densen [48,49] when they analysed polytene chromosome patterns, provided evidence for genetically distinct *An. cruzii *populations. They showed that three X chromosome forms (A, B and C) exist, suggesting a process of incipient speciation of this species. As heterozygous individuals were never found in these populations, the authors discarded a hypothesis of chromosome polymorphism.

The possibility that *An. cruzii *may be a complex was supported by isoenzymatic analysis of several *An. cruzii *populations. *An. cruzii *populations in southern and southeastern Brazil (in the states of Santa Catarina, São Paulo and Rio de Janeiro) appear to be closely related to each other but genetically distinct from a population in the south of the state of Bahia [50]. It appears that there is a continuous area in southern Brazil in which one species is distributed, and that this is separated from a different population found in the south of the state of Bahia.

Recently, Malafronte *et al *[51] compared sequences of the second internal transcribed spacer of ribosomal DNA (ITS2) of several *An. cruzii *populations with the chromosomal forms proposed by Ramirez and Densen [48,49]. Their findings showed high levels of ITS2 sequence polymorphism among *An. cruzii *captured in the states of São Paulo and Santa Catarina. However, in contrast to the results obtained by Malafronte *et al *[51], restriction digest analysis of ITS2 fragments showed no polymorphism in banding patterns among an F1 generation of *An. cruzii *mosquitoes captured in the states of Santa Catarina, Paraná and São Paulo [52]. Although the results were not conclusive, all the studies strongly suggest that *An. cruzii *is a complex of at least two sibling species. Calado and Navarro-Silva [53] identified specific digestion patterns in *An. cruzii *and *An. homunculus *using ITS2 RFLP, indicating that this could be used as a tool to distinguish between these species. It should be noted that none of the ITS2 sequences generated by Calado *et al *[52] and Calado and Navarro-Silva [53] have been deposited in GeneBank, preventing further analyses of the sequences used in both studies.

Among the remaining *Kerteszia *species, this approach was only used with *An. bellator *species, and the results indicated a low level of gene flow between Brazilian populations.

When Brazilian and Trinidad populations were compared, gene flow between populations was even lower [54]. The authors could not explain the apparent interruption in the geographical distribution of this species in North and South America and suggested that a recent geographical split of *An. bellator *populations may not have allowed enough time for speciation.

## Conclusion

The *Kerteszia s*pecies Atlantic forest domain are relatively well known in Brazil. However, the distribution limits of each species need to be studied in detail because of the scarcity of distribution studies and the continuous and intense environmental changes that can restrict the geographic distribution of a mosquito taxon.

No larval studies in natural habitats have been carried out in recent years, and these should be encouraged. Studies of the general activity of *Kerteszia *anophelines, particularly that of adult females, could explain the epidemiological role of the main vector species involved in the propagation of human and simian malaria parasites. However, the parasitological aspect of transmission is still rarely explored in scientific works, and many questions remain to be answered. These include maintenance of autochthonous malaria, the zoonotic nature of the parasitism, the role played by monkeys and the introduction of parasites from other areas.

Traditional taxonomy can clearly distinguish this subgenus and its respective species, and reliable means for identifying external morphology are available. However, molecular taxonomy has given rise to new challenges by showing the existence of population variations at the genomic level. These variations suggest the existence of sibling species complexes in the distribution area of *An. cruzii*. Detailed morphological studies are required to evaluate whether there are morphological variations corresponding to the molecular variations observed in populations of *An. cruzii*. Studies of the vector competence and capacity of these possible cryptic species are also needed.

To this end, training in both species identification and field work is crucial to develop a reliable molecular taxonomy. Analysis of F1 progenies from wild mosquitoes is needed not only for the *Kerteszia *subgenus but in all studies involving molecular and phylogenetic analysis, as some phenotype characteristics are only seen in one gender (male or female) depending on the mosquitoes that are being identified. Additionally, analysis of F1 progeny will allow a better evaluation of both individual morphological and molecular variation and will enable voucher sequences generated from a species to be kept.

The tendency is for the territory of the *Kerteszia *subgenus in Brazil, and thus the populations of these anophelines, to be preserved because of the growing environmental protection as a result of worldwide ecological awareness. This can be explained by the strong dependence of this taxon on the forest, and thus the terms "forest-malaria" and "bromeliad-malaria" are both appropriate.

With a low incidence of malaria, the current epidemiological picture is expected to be maintained in the future by either zoonotic transmission or the introduction of *Plasmodium *from endemic regions to risk areas. However, occasional malaria outbreaks have happened in the area in the last years, with significantly higher number of malaria cases compared with what was being annually reported, probably due to environment changes and the high number of asymptomatic individuals in the area acting as reservoirs. In this way, all the territory in the Atlantic forest that survived deforestation will need to be carefully monitored, not only by researchers but also by health and control services. As environmental legislation covering these areas comes into effect, there will be a tendency for malaria transmission to persist in this region.

## Authors' contributions

DN conceived and wrote the first draft of the article. MTM helped with its design and content and with coordination of the draft manuscript. MAMS and RSM contributed to the structure and content and were involved in re-drafting the article. All the authors read and approved the final manuscript
